# Failure of recombinant factor VIIa in a patient with severe polymicrobial sepsis and postoperative uncontrolled intraabdominal bleeding

**DOI:** 10.1186/1471-2334-7-34

**Published:** 2007-04-26

**Authors:** Anna Conen, Maja Weisser, Dimitrios A Tsakiris, Martin Siegemund

**Affiliations:** 1 Department of Infectious Diseases, University Hospital, Basel, Switzerland; 2 Medical Intensive Care Unit, University Hospital, Basel, Switzerland; 3 Division of Haematology, University Hospital, Basel, Switzerland; 4 Department of Anaesthesia and Intensive Care Medicine, University Hospital, Basel, Switzerland

## Abstract

**Background:**

This report discusses a case of unsuccessful treatment with recombinant factor VIIa (rFVIIa) in off-label use. The need for international guidelines concerning the off-label use of rFVIIa is outlined as well as the need for methods to control the efficacy of rFVIIa objectively.

**Case presentation:**

54 year old male with severe polymicrobial sepsis due to a perforated diverticulitis of the sigmoid colon and consecutive overt disseminated intravascular coagulation. He suffered severe intraabdominal bleeding after abdominal surgery despite conventional haemostatic support. Repeated applications of factor VIIa temporarily improved coagulation essays but did not stop clinical bleeding. The patient died in multiorgan failure due to septic and haemorrhagic shock.

**Conclusion:**

Off-label use of rFVIIa could result in more side effects than could be expected from literature because of a publication bias. However for most off-label applications large prospective, randomised and controlled trials to confirm the positive findings are missing. For the future, not only guidelines concerning the off-label use of rFVIIa are urgently needed but also guidelines for monitoring the efficacy of rFVIIa.

## Background

Recombinant factor VIIa (rFVIIa) has been used since 1988 to control bleeding in haemophilia A and B patients with inhibitors against factor VIII and IX [1-3]. A standard dose of 90 μg/kg body weight in repeated administrations was defined as being efficacious. This is the only internationally licensed indication for rFVIIa. However, the agent became famous for being successful in other critical situations, where uncontrolled bleeding persisted despite massive conventional haemostatic support. Successful treatments were reported in patients with inherited factor VII deficiency or with thrombocytopathy, upper gastrointestinal bleeding, after surgery or trauma, in bleeding patients treated with anticoagulants (warfarin, tirofiban) and in patients with disseminated intravascular coagulation (DIC). These indications are known in the literature as off-label use and are based on data published as case reports or observational studies, almost always demonstrating a beneficial effect [4]. Off-label use is described in national guidelines [5,6], which are worked out mostly by haematologists and specialists in intensive care medicine. Only a few prospective randomised trials exist in non-haemophilic patients examining the indication, dose regimen, efficacy and safety of the treatment with rFVIIa [7-10]. In these trials a positive effect of rFVIIa was shown in patients with intracerebral haemorrhage (limited growth of the haematoma and reduced mortality) [7] and in patients suffering a blunt trauma (reduced transfusion requirements) [8]. Only a trend toward a better outcome was shown in patients with a penetrating trauma receiving rFVIIa. On the other hand, no positive effect of rFVIIa was documented in patients after haematopoietic stem cell transplantation (HSCT) suffering a bleeding complication from any cause [9], or in patients undergoing an orthotopic liver transplantation in end-stage liver disease [10].

rFVIIa acts at sites where the endothelium is damaged and tissue factor is released, implying a local mode of action. rFVIIa forms a complex with the tissue factor and then activates factor X and platelets, resulting in the local generation of thrombin and consecutively in the formation of a stable fibrin clot [11].

We describe a severely ill patient suffering from overt DIC, who received rFVIIa because of uncontrolled intraabdominal bleeding after abdominal surgery. The patient died in septic and haemorrhagic shock despite adequate treatment and use of rFVIIa (Novoseven^®^).

## Case presentation

**Figure 1:**
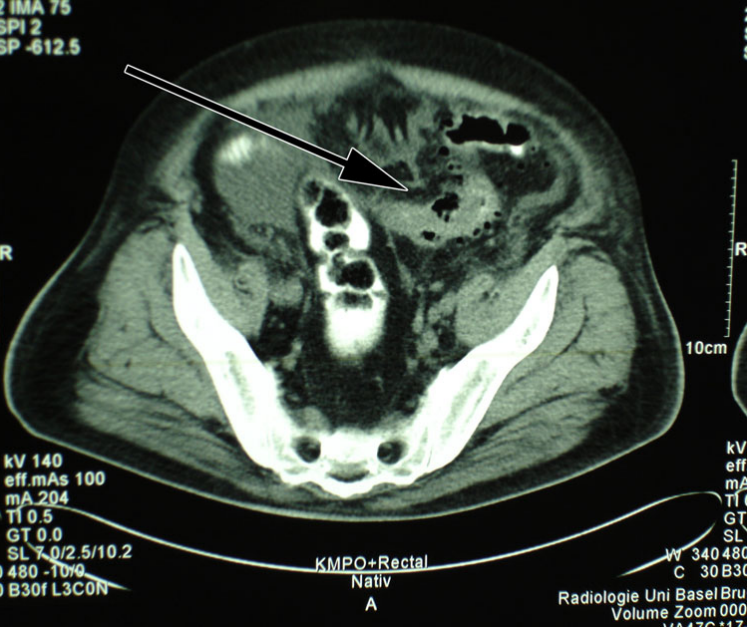
Perforated diverticulitis of the sigmoid colon.

**Figure 2:**
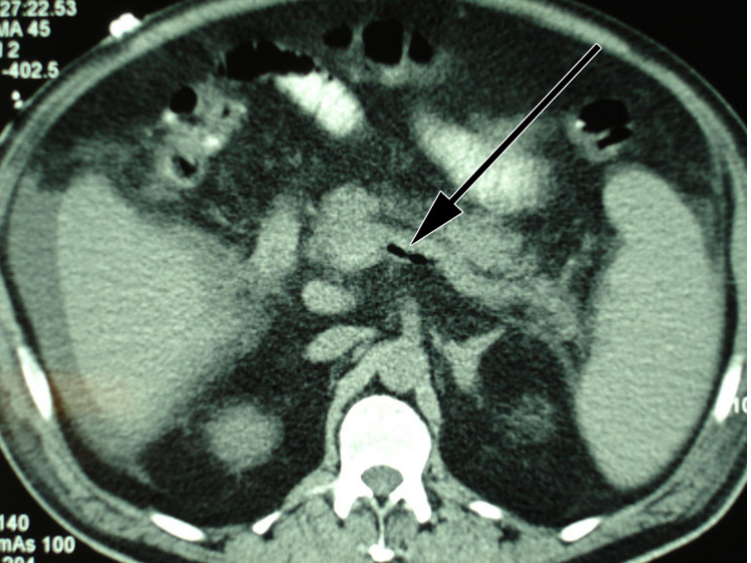
Air in the inferior mesenteric vein.

**Figure 3:**
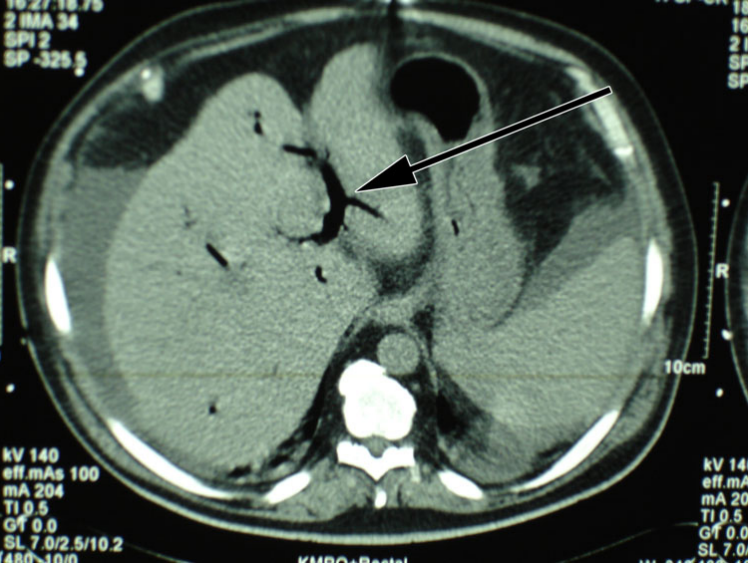
Air in the portal vein system.

A 54 year old man presented in the emergency department of our hospital with a three weeks history of fever, shivering, abdominal discomfort and watery, unbloody diarrhoea. The patient's medical history was otherwise remarkable for a metabolic syndrome with arterial hypertension, diabetes mellitus type 2 and obesity. His regular medication consisted of metformin and enalapril/hydrochlorothiazide. The clinical examination confirmed a bad general health condition with intermittent fever up to 40°C and shivering. He was hypotensive (85/55 mmHg), had a normal pulse rate and was tachypnoic (20 breaths/min.). The abdomen was distended; showed normal bowel sounds and rebound tenderness – consistent with a diffuse peritonism. Furthermore the clinical examination was unremarkable. Laboratory profile was consistent with systemic inflammation, acute renal failure and hepatopathy. Platelets were low with 41 G/l (normal range 150–450 G/l). Lactate and coagulation parameters were within normal limits. Antibodies for hepatitis A, B and C were negative. Urine analysis was unremarkable. The abdominal computerized tomography (CT scan) showed a big retroperitoneal abscess provoked by a diverticulitis of the sigmoid colon, as well as air in the portal vein system and in the inferior mesenteric vein (pylephlebitis). Ascites and hepatosplenomegaly were present (figure 1, 2, 3).

Sepsis due to the described intraabdominal focus was diagnosed and a broad-spectrum antibiotic treatment with intravenous piperacillin/tazobactam and amikacin was started. After administration of fluids blood pressure stabilized. Cultures of ascites fluid were positive for Klebsiella pneumoniae, Escherichia coli and Candida albicans. In blood cultures Escherichia coli, Klebsiella oxytoca and Bacteroides fragilis could be isolated without resistances against piperacillin/tazobactam. In order to treat the intraabdominal growth of Candida albicans high-dose fluconazole was added. The patient was operated on the second hospital day to evacuate the retroperitoneal abscess (Hartmann's operation: sigma resection, generating a colostomy with the proximal end of the descending colon and a blind rectal pouch with the proximal end of the distal segment). Because of anuria haemofiltration was started after surgery. During haemofiltration low doses of unfractionated heparin were administered to keep the filter patent.

**Figure 4:**
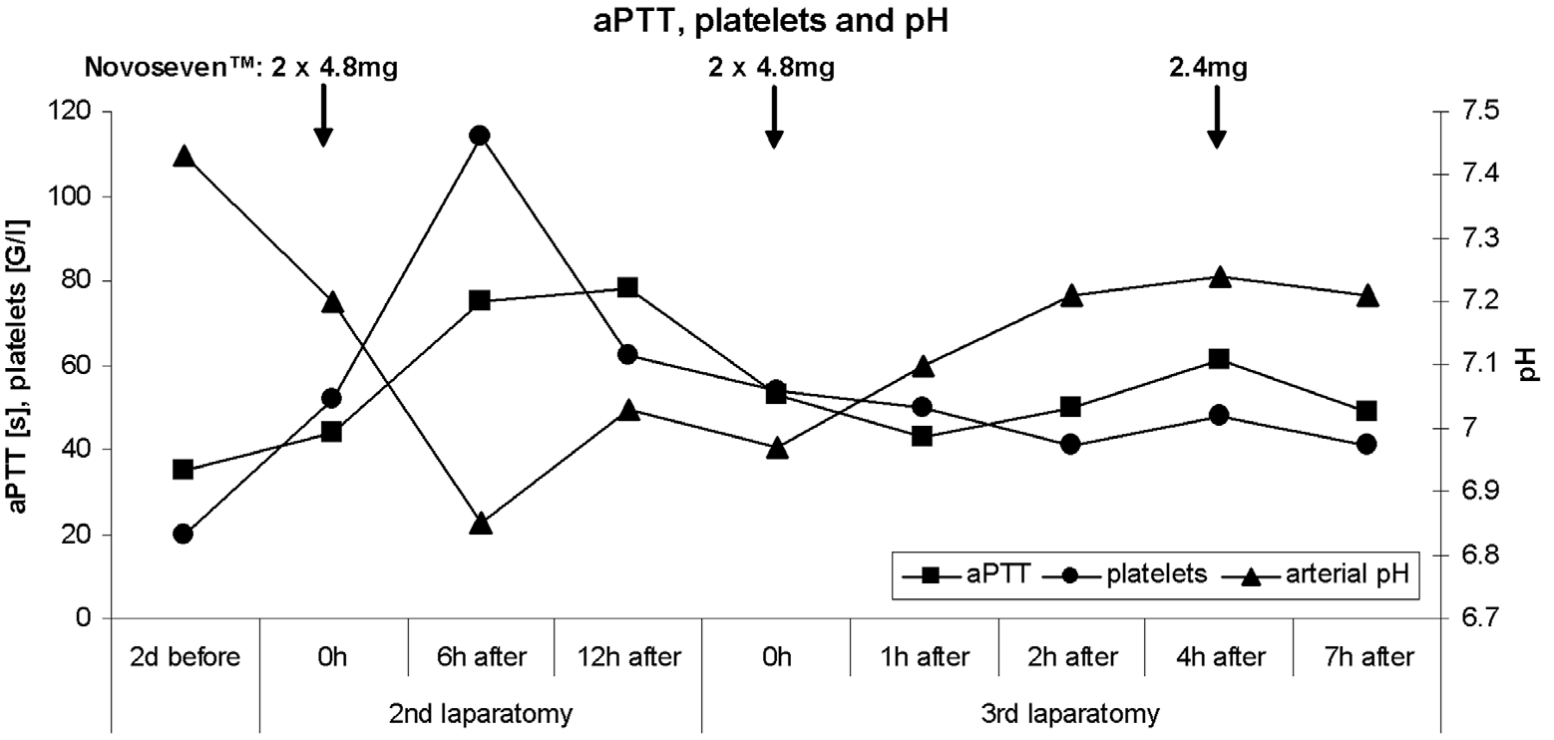
Measured values of aPTT, platelets and arterial pH during the treatment with rFVIIa.

One week later – on day 9 of hospitalisation – secretions from abdominal drainages became bloody and cloudy with an amount of 2000 ml/day. The coagulation profile at this time was: thrombocytes 52 G/l, international normalized ratio (INR) 1.3 (normal range <1.3), prothrombin time (PT) 14.5 s (normal range <11 s), activated partial thromboplastin time (aPTT) 44 s (normal range 25–34 s), fibrinogen 3.2 g/l (normal range 1.7–4 g/l) and factor VII 29% (normal range 70–120%). There was a progressive metabolic acidosis with lactate levels around 6 to 17 mmol/l (normal range <1.8 mmol/l). As the treatment with metformin had been stopped at admission, when lactate levels were within normal limits, the current progressive lactate acidosis was ascribed to ongoing sepsis. With raising inflammatory markers, antibiotic therapy was changed to meropenem and a second laparotomy was performed. A putrid retroperitoneal haematoma was removed. During surgery, diffuse intraabdominal bleeding required extended surgical blood coagulation and the substitution of 1 platelet (TC) and 6 packed red cell concentrates (RC), as well as 7 units of fresh frozen plasma (FFP). At this time the patient received intraoperatively one dose of rFVIIa (70 μg/kg). The coagulation profile at this time was consistent with a DIC with platelets 62 G/l, INR 1.4, PT 16 s, D-Dimers 3.22 μg/ml (normal range <0.5 μg/ml), fibrinogen 1.2 g/l and aPTT 80 s. Because of the sustained bleeding the abdomen was left open to prevent intraabdominal compartment syndrome. Postoperatively high doses of vasoactive drugs had to be administered to maintain circulation. Severe metabolic acidosis (pH 6.9) with a lactate of 22 mmol/l was present because haemofiltration had to be discontinued during surgery. The bleeding remained uncontrolled despite further substitution of 1 TC and 6 RC, 5 FFP, tranexamic acid (Cyclokapron^®^), protamin hydrochloride (Protamin^®^) and von Willebrand factor concentrate (Haemate^®^). Therefore, a second dose of rFVIIa (70 μg/kg) was given. Twelve hours after the second laparotomy – in the morning of day 10 – a third laparotomy was performed. Again, a haematoma with diffuse retroperitoneal bleeding had to be removed. Surgical haemostasis and retroperitoneal cloth packing for tamponade were performed. The patient received intraoperatively 4 EC, 6 FFP and 2 doses of rFVIIa (each 70 μg/kg). After the surgery, the bleeding remained uncontrolled despite the administration of 3 FFP every 8 hours, rFVIIa every 4 hours (35 μg/kg) and TC and RC as needed (figure 4). The patient died 15 hours after the third operation in multiorgan failure. No autopsy was performed.

## Discussion

We described a patient with severe polymicrobial sepsis secondary to a perforated diverticulitis of the sigmoid colon with pylephlebitis, who developed an overt DIC and uncontrolled intraabdominal bleeding postoperatively. Despite massive transfusions and the substitution of haemostatic agents including rFVIIa, bleeding persisted and the patient died in septic and haemorrhagic shock 10 days after being hospitalised.

Many case reports describing clinical efficacy and safety of rFVIIa in different acquired bleeding situations can be found in literature [4]. Despite these positive experiences, randomised and controlled trials are rare [7-10]. But those trials would be crucial for developing international guidelines concerning the off-label use of rFVIIa. Questions as "who should get rFVIIa at what time in what dose?" need to be answered.

There are known factors negatively influencing the efficacy of rFVIIa (e.g. hypothermia, acidosis, prolonged preadministration prothrombin time or hyperfibrinolysis) [12]. These factors should be corrected prior to the administration of rFVIIa. Especially acidosis seems to compromise the enzymatic actions in the coagulation cascade. Meng et al [13] describe that a decrease in pH from 7.4 to 7.0 reduces the activity of rFVIIa by 90% in an in vitro setting. However, the optimal time point of administration of rFVIIa is difficult and not well defined yet: administering rFVIIa too early may decrease efficacy due to insufficient substitution of red blood cells, platelets and antifibrinolytics, but also due to initial acidosis; waiting too long may worsen metabolic state, aggravate acidosis and therefore coagulopathy. In a retrospective analysis by Clark et al [14], examining rFVIIa as a "last-ditch" in patients with massive haemorrhage unresponsive to conventional pharmacologic and surgical treatment and to massive transfusions, high mortality rates (1-day and 7-day mortality 40% and 70% respectively) were noted with only 60% of the patients receiving rFVIIa responding at least transiently with a cessation of bleeding. The authors conclude that last-ditch rFVIIa therapy is ineffective in patients who do not respond to extended conventional treatment. Our patient received rFVIIa as a last ditch therapy when overt DIC with prolonged PT (16 s) and elevated D-Dimers (3.22 μg/ml) was already present and massive transfusions were unsuccessful. He was in a severe metabolic acidosis (pH 6.9 and lactate >20 mmol/l) at the time of administration. All factors together – prolonged preadministration prothrombin time, hyperfibrinolysis and especially severe acidosis – might be causal for unresponsiveness to rFVIIa, as might be the progressive liver failure.

The use of rFVIIa in patients with DIC – where there is systemic activation of coagulation with systemic exposure of tissue factor [15] – could lead to more pronounced side effects. But no controlled trials exist to confirm this hypothesis. Case reports and case series show successful use of rFVIIa in DIC [16-18]. Noteworthy is that no thromboembolic complications occurred in those cases. However, a publication bias can not be excluded. It is difficult to compare the published cases with our patient concerning the effectiveness of rFVIIa. First we state that doses of rFVIIa administered were different, 90 μg/kg versus 70 μg/kg or less in our patient; second, in the published cases there is no information on the metabolic situation and worsened action of rFVIIa by metabolic acidosis. However, in our patient the metabolic situation with severe acidosis might explain at least partially the lack of effect.

Ineffectiveness of rFVIIa has not been published very often so far in case reports [19], but there are three randomized controlled trials, in which rFVIIa did not show any benefit compared to placebo [8-10]. In the latter two cited studies different doses of rFVIIa were administered (20, 40 or 80 μg/kg, respectively 40, 80 or 160 μg/kg). In the literature no consensus exists about the appropriate dosage of rFVIIa. Used dosages range from 15–160 μg/kg in single or multiple administrations, making comparisons difficult.

Major side effects of rFVIIa are thromboembolic events (myocardial infarction, cerebrovascular accidents, pulmonary embolism, thrombosis in other arterial or venous sites and clotted devices). Other, non thromboembolic adverse events are rash, allergic reactions, lack of effect and unspecific symptoms such as pain, nausea or vomiting [20,21]. Sometimes it is difficult to relate the reported event to rFVIIa, because some events occur days after the administration or occur in patients with severe co-morbidities, offering a more probable explanation for a thromboembolic event than rFVIIa.

Besides clinical responsiveness, the effect of rFVIIa should be monitored with laboratory parameters, whereas appropriate tests are discussed controversially. Thromboelastography would allow the evaluation of the effects of rFVIIa on haemostasis [22,23]. This method consists of a viscoelastic measurement of clotting time, clot formation time and maximum clot firmness in whole blood, but it is not routinely available in most hospitals. The conventional measurement of the activity of factor VII or VIIa in plasma after the administration of rFVIIa only proves the existence of factor VII and its successful administration, but it does not predict any effect on haemostasis [11,22]. The factor VII activity in our patient, measured with conventional methods, was consistently >120% after the repetitive administration of rFVIIa. This was reflected in a transient shortening of aPTT- and INR-values (figure 4). Despite this in vitro activity, factor VIIa was not clinically effective in our patient. The only conclusion allowed by the conventional measurement therefore is that the drug was successfully administered intravenously. The objective parameters measured by thromboelastography would have been helpful not only in controlling efficacy of rFVIIa but also in dose finding decisions. For the future, hence, not only guidelines concerning the off-label use of rFVIIa are urgently needed but also guidelines for monitoring the efficacy of rFVIIa.

## Conclusion

In many case reports, efficacy and safety of rFVIIa in the off-label use are discussed. For most off-label applications large prospective, randomised and controlled trials are however missing, confirming the positive findings. Based on such trials, international guidelines should be elaborated, giving consent on how and when to administer rFVIIa. The off-label use of rFVIIa could result in more side effects than could be expected from the literature because of a publication bias. On the other hand, it also implies high costs, which nowadays are generally a major issue in medicine. For the future, not only guidelines concerning the off-label use of rFVIIa are urgently needed but also guidelines for monitoring the efficacy of rFVIIa.

## Key messages

• Guidelines for the off-label use of rFVIIa are required.

• Guidelines for monitoring the efficacy rFVIIa are required.

• The efficacy of rFVIIa could be overestimated because of a potential publication bias.

## Abbreviations

rFVIIa – recombinant factor VIIa

DIC – disseminated intravascular coagulation

HSCT – haematopoietic stem cell transplantation

CT scan – computerized tomography

INR – international normalized ratio

PT – prothrombin time

aPTT – activated partial thromboplastin time

TC – platelet concentrate

RC – packed red cell concentrate

FFP – fresh frozen plasma

## Competing interests

The author(s) declare that they have no competing interests.

## Authors' contributions

AC summarized the case and designed and drafted the manuscript. MW participated in the design and coordination of the manuscript. DT revised the manuscript to be theoretically sound. MS initialized the case report and helped to draft the manuscript. All authors read and approved the final manuscript.

## Pre-publication history

The pre-publication history for this paper can be accessed here:


